# Governing at speed? China’s hard question in need of soft solutions

**DOI:** 10.1016/j.omtn.2026.103096

**Published:** 2026-09-25

**Authors:** Joy Y. Zhang

**Affiliations:** 1 University of Kent, Centre for Global Science and Epistemic Justice, Canterbury, Kent CT1 7NZ, UK

## Main text

China is one of the world’s most exciting sites of biomedical innovation, not only because it has emerged as a global scientific powerhouse—ranking first in biological sciences and second in health sciences^1^—but also because its regulators have been willing to experiment with new regulatory pathways, creating space for early-stage clinical exploration and responses to unmet medical needs.^2^ Yet, China may also be particularly vulnerable to poorly governed scientific experimentation undermining public confidence and triggering backlash, both domestically and globally. The hard question is: can China develop governance capacity at the same pace?

Two recently exposed deaths in gene therapy trials bring this question into sharp focus. A 6-year-old girl, Mei, with a rare neurodevelopmental disorder, died 7 days after receiving an experimental adeno-associated virus (AAV)-delivered base-editing therapy in an investigator-initiated trial at Xinhua Hospital, affiliated with Shanghai Jiao Tong University.^3^ Less than 2 weeks after this case was reported, *STAT News* revealed another previously undisclosed child’s death—a boy with Duchenne muscular dystrophy who died after receiving a high dose of HG302, an experimental gene therapy developed by Shanghai-based HuidaGene, also through an investigator-initiated trial.^4^ For many observers, the two deaths immediately recalled the 2018 “CRISPR babies” scandal—the illegal use of heritable human genome editing by researcher-entrepreneur He Jiankui, which resulted in the birth of three gene-edited girls.^5^

However, as someone who has studied China’s life science governance for more than two decades and was also involved in Chinese academic communities’ response to the “CRISPR babies” scandal,^5^^,^^6^ I find these recent cases more worrying. I want to draw attention to three points, both to place these cases in the context of China’s rapid scientific development and to consider what they reveal about how the credibility and robustness of its governance could be strengthened.

The first key difference is institutional. The “CRISPR babies” scandal involved a scientist at a then relatively young university, working partly during his sabbatical and flagrantly violating existing Chinese clinical and ethical regulations. The two recent cases, by contrast, were investigator-initiated studies that received institutional authorization within China’s IIT governance system and were subject to the 2024 Measures for the Management of Investigator-Initiated Clinical Research in Medical and Health Institutions, which came into force in October 2024.^7^ While the 2018 scandal could be characterized as the work of a “rogue” scientist and was swiftly condemned by the mainstream Chinese scientific community, ironically including the Principal Investigator involved in the Shanghai Jiaotong University case, the recent cases raise a harder question: what happens when ethical governance fails from within established scientific institutions themselves?

Secondly, and perhaps most worryingly, the two cases emerged after the Chinese government had introduced a slew of policies in response to the “CRISPR babies” scandal aimed at strengthening ethical governance, including explicit commitments to build the capacity of institutional ethics review boards.^5^^,^^8^ It is also significant that both cases reported in 2026 occurred in Shanghai, a municipality with a historically well-connected and internationally informed scientific community that has been at the forefront of ethical debate and the institutionalization of ethics review in China, as demonstrated by its earlier experience with stem cell research and related experimental therapies.^6^

What is most troubling about the Shanghai Jiaotong University case, however, is not simply that Xinhua Hospital’s ethics committee approved the clinical trial without reviewing the final report of the primate safety study but that after the girl’s death, the committee concluded that her death was “definitely related” to the treatment,^3^ yet public recruitment for the experiment was allowed to continue. This points to a deeper problem. Despite the Chinese authorities’ commitment to strengthening the institutional ethical governance, ethics review boards, even in a leading scientific hub such as Shanghai, may remain marginal to research decision-making, with limited authority once research is under way. The hard question is, therefore, not simply whether appropriate rules and review mechanisms exist, but whether those charged with ethical governance have the authority and resources to make them matter.

Thirdly, both tragedies were exposed by foreign media roughly a year after the deaths occurred. In the Shanghai Jiaotong University case, Mei’s parents reportedly asked for the research findings to be retracted to avoid misleading other families with similarly affected children, but their request went unheeded. In the HuidaGene case, the company’s former CEO and co-founder Alvin Luk said that he had pushed for details of the fatality to be disclosed, also without success.^3^^,^^4^ The two cases raise an uncomfortable question: could political pressure to contain “bad news” in the name of social stability, combined with an intensely competitive scientific culture that rewards success, makes failure unusually difficult to disclose?

However, it would be wrong to respond to these cases by dismissing either the Chinese scientific community or the value of experimenting with new regulatory pathways. The deeper problem is one of imbalance. China has invested enormously in scientific capacity and increasingly in the formal architecture of ethical governance but not proportionately in empowering its ethics community or building the infrastructure through which failures can be disclosed, scrutinized, discussed, and learned from. Without the necessary cultural change and resources, regulatory flexibility risks accelerating experimentation without developing an equivalent capacity for collective learning and harm reduction.

There is an important irony here. In November 2025, researchers from Nanjing University argued in a *Nature* correspondence that “China should undertake more risky research to close the Nobel gap.”^9^ Despite its provocative title, their argument was not a call for reckless experimentation. Quite the opposite: they criticized a research system oriented toward short funding cycles, fragmented projects, and quick returns and called for sustained support for ambitious, long-term research. Read in this light, their argument speaks directly to one of the problems exposed by the Shanghai cases: a short-termism that rewards quick results and individual competitiveness without building the institutional capacity for transformative scientific advances and their responsible translation.

China’s State Council Order No. 818, promulgated last September and in force since May 2026, introduced a higher-level, dedicated regulatory regime for “new biomedical technologies,” including technologies acting at the cellular and molecular levels, and explicitly connected clinical research with clinical translation.^10^ Yet, the Shanghai cases illustrate why governing at speed cannot be achieved through formal regulation alone. Gene therapy makes this tension particularly visible. The answer may lie in “soft solutions” to hard governance problems: giving ethics communities genuine authority; creating protected spaces where adverse outcomes and failures can be reported, debated, and learned from; and rewarding long-term collective achievement rather than short-term individual success. The challenge is not to curb scientific ambition but to create the conditions for pursuing it responsibly.

## Declaration of interests

The author declares no competing interests.

## References

[1] Nature Index (2026). Research Leaders: Leading countries/territories in health sciences. Nature Index.

[2] Yin C., Gao J., Li G., Hu H., Zhou L., Lu S., Chen X. (2022). Gene and cell therapies in China: booming landscape under dual-track regulation. J. Hematol. Oncol..

[3] Borrell B., Retraction W. (2026). Exclusive: Death of girl in Chinese gene-editing trial was never made public. Science.

[4] Mast J., Yang B. (2026). Once Again, Child Dies in Gene-Editing Trial in China, Rekindling Debate on Transparency and Safety. STAT News.

[5] Zhang J.Y., Lei R. (2023). Is Chinese bioethics ready to move forward from the CRISPR baby scandal?. Hastings Bioethics Forum.

[6] Zhang J.Y. (2012). https://www.palgrave.com/gb/book/978023030.

[7] National Health Commission, National Administration of Traditional Chinese Medicine, and National Disease Control and Prevention Administration (2024). Measures for the Management of Investigator-Initiated Clinical Research in Medical and Health Institutions. https://www.gov.cn/zhengce/zhengceku/202409/content_6976872.htm.

[8] Liu Z., Shi J., Xu J. (2024). He Jiankui’s unprecedented offense and worrying comeback: how the CRISPR-babies scandal reshaped the legal governance of scientific research in China. J. Responsible Innov..

[9] Yang A.J., Deng S., Freeman R.B. (2025). China should undertake more risky research to close the Nobel gap. Nature.

[10] State Council of the People’s Republic of China (2025). Regulation on the Administration of Clinical Research and Clinical Translational Application of New Biomedical Technologies. State Council.

